# Quality of Life determinants in women with breast cancer undergoing treatment with curative intent

**DOI:** 10.1186/1477-7819-3-63

**Published:** 2005-09-27

**Authors:** Manoj Pandey, Bejoy Cherian Thomas, Padmakumar SreeRekha, Kunnambath Ramdas, Kuttan Ratheesan, Sankarannair Parameswaran, Beela S Mathew, Balakrishnan Rajan

**Affiliations:** 1 Departments of Surgical Oncology, Regional Cancer Centre, Trivandrum, India; 2 Department of Radiation Oncology, Regional Cancer Centre, Trivandrum, India; 3 Department of Surgical Oncology, Jawaharlal Nehru Cancer hospital and Research Centre, Bhopal, India

## Abstract

**Background:**

The diagnosis of breast cancer and its subsequent treatment has significant impact on the woman's physical functioning, mental health and her well-being, and thereby causes substantial disruption to quality of life (QOL). Factors like patient education, spousal support and employment status, financial stability etc., have been found to influence QOL in the breast cancer patient. The present study attempts to identify the determinants of QOL in a cohort of Indian breast cancer patients.

**Patients and methods:**

Functional Assessment of Cancer Therapy-Breast (FACT-B) Version 4 Malayalam was used to assess quality of life in 502 breast cancer patients undergoing treatment with curative intent. The data on social, demographic, disease, treatment, and follow-up were collected from case records. Data was analysed using Analysis of Variance (ANOVA) and multinomial logistic regression.

**Results:**

The mean age of the patients was 47.7 years with 44.6% of the women being pre-menopausal. The FACT-B mean score was 90.6 (Standard Deviation [SD] = 18.4). The mean scores of the subscales were – Physical well-being 19.6 (SD = 4.7), Social well-being 19.9 (SD = 5.3), Emotional well-being 14 (SD = 4.9), Functional well-being 13.0 (SD = 5.7), and the Breast subscale 23.8 (SD = 4.4). Younger women (<45 years), women having unmarried children, nodal and/or metastatic disease, and those currently undergoing active treatment showed significantly poorer QOL scores in the univariate analysis. However multivariate analysis indicated that the religion, stage, pain, spouse education, nodal status, and distance travelled to reach the treatment centre as indicative of patient QOL.

**Conclusion:**

QOL derangements are common in breast cancer patients necessitating the provisions for patient access to psychosocial services. However, because of the huge patient load, a screening process to identify those meriting intervention over the general population would be a viable solution.

## Background

Breast cancer is the leading cause of cancer death among women around the world. In India it shows mix incidence pattern with breast cancer being second to cancer of the cervix in rural areas [1,2], however, in metropolitan cities like Mumbai, New Delhi and Trivandrum, the incidence of breast cancer has crossed that of cervix. The incidence of breast cancer in India ranges from 8.8/100,000 at Barshi to 28.6/100,000 at Mumbai [2]. In Trivandrum, the age-adjusted-rate (AAR) is 31.7/100,000 for the urban population and 16.5/100,000 for rural population [3].

The focus of breast cancer care, in addition to examining short-term treatment related quality of life (QOL) outcomes, has expanded to include acute treatment-related side effects and long-term factors that influence the quality as well as quantity of survival [4-7]. Considerable efforts are directed to reduce morbidity from treatment and rehabilitation. Scenario in India is little different. In absence of screening programmes, majority of the breast cancers are still diagnosed in locally advanced stage and achieving longer survival is still a priority. A few studies on QOL in the Indian context exist, factors like patient education, spousal support and employment status, financial stability, disease stage, etc., have been found to influence patient QOL [8,9].

QOL domains like levels of physical, social, and psychological well-being have been found to be comparable to those of women without the disease [10,11]. Initially, women with breast cancer, especially younger women, tend to suffer substantial disruption in their physical functioning, mental health and well-being [12,13]. Due to this wide variability in QOL [14,15] identification of factors that render women vulnerable to negative outcomes and poor QOL is essential [6]. This study aims at identifying the determinants of QOL of Indian women with breast cancer treated with curative intent, on a cross-sectional cohort of patients interviewed at a single cancer care centre.

## Patients and Methods

The study sample consisted of 504 breast cancer patients who were undergoing or had undergone curative treatment at our centre. The tool was administered either at the beginning of the treatment or at follow-up after the treatment. The earlier validated local language version [8] of the Functional Assessment of Cancer Therapy-Breast, Version 4 (FACT-B) [16] was used. FACT-B is a 36 item self administered scale containing 4 general subscales i.e. physical, social/family well being, functional and emotional well being, the fifth subscale contain 9 items and is specific for breast cancer. Written consent was obtained from all the patients prior to administering the tool. The study was approved by the Institutional research board and the Ethics committee. The test was administered and scored in accordance with the instructions in the manual for the Version 4 of the Functional Assessment of Chronic Illness Therapy (FACIT) Measurement System [17]. Group comparisons were carried out by using one-way analysis of variance (ANOVA). Multivariate analysis was carried out using multiple logistic regression, the data was dichotomised using the median value and factors identified by literature search, and univariate analysis were entered into the model in single step (step method).

## Results

Mean age of the patients was 47.6 years (SD = 11, range 20–80, median 47 years). Of the 502 patients almost equal number belonged to upper, middle and lower class (Table 1). Majority of the patients were Hindus (78%) resided within 150 km of the centre and most were married (75%). Other population characteristics are described in table 1.

**Table 1:** Frequency and percentage distribution of demographic characteristics of study population

**Variable**	**Grouping Codes**	**Group**	***N***	**Percentage**
**Interviewer**	1	BCT	197	39.2%
	2	SR	305	60.8%
**Social class**	1	Low	167	33.3%
	2	Middle	164	32.7%
	3	High	171	34.1
**Distance to centre (Km)**	1	Local	150	29.9%
	2	<150	241	48.0%
	3	<150–250	61	12.2%
	4	>250	49	9.8%
	5	Don't know	1	0.2%
**Religion**	1	Hindu	323	64.3%
	2	Muslim	71	14.1%
	3	Christian	94	18.7%
	9	Others/Don't know	13	2.6%
**Marital status**	1	Single	23	4.6%
	2	Married	377	75.1%
	3	Widow/Divorce	100	19.9%
**Self education**	1	Illiterate	23	4.6%
	2	≤5	96	19.1%
	3	6–10	255	50.8%
	4	11–12	55	11.0%
	5	Graduate/tech	40	8.0%
	6	Post graduate	29	5.8%
**Spouse education**	1	Illiterate	12	2.4%
	2	≤5	64	12.7%
	3	6–10	198	39.4%
	4	11–12	26	5.2%
	5	Graduate/tech	44	8.8%
	6	Post graduate	31	6.2%
	7	Don't know	127	25.3%
**Self Occupation**	1	HW/Unemployed	380	75.7%
	2	Employed	81	16.1%
	3	Self/Business/Daily	31	6.2%
	9	Don't know	10	2.0%
**Spouse Occupation**	1	HW/Unemployed	50	10%
	2	Employed	135	26.9%
	3	Self/Business/Daily	170	33.9%
	9	Don't know	146	29.1%

Over 90% of the patients had been diagnosed prior to being referred to tertiary centre for treatment and 22% of them had underwent surgery in form of either modified radical mastectomy or breast conservation elsewhere (Table 2). Most of the patients had T_2 _disease (34.7%) followed by T_3 _(16%) and T_4 _(15%). Axillary nodes were present in 42% of the sample (Table 2).

**Table 2:** Frequency and percentage distribution of disease characteristics in patients

Variable	Grouping Codes	Group	*N*	Percentage
Symptoms	1	Lump	426	84.9%
	2	Ulcer	3	0.6%
	3	Discharge	28	5.6%
	9	Don't know	44	8.8%
Pain	1	Yes	133	26.5%
	2	No	334	66.5%
	3	Don't know	35	7.0%
How diagnosed	1	Biopsy	212	42.2%
	2	FNAC	239	47.6%
	3	Mammogram only	4	0.8%
	9	Don't know	46	9.2%
Previous Treatment	0	Nil	218	43.4%
	1	Excision	84	16.7%
	2	MRM	110	21.9%
	3	BCT	2	0.4%
	9	Don't know	88	17.6%
Tumour staging	1	T_1_	33	6.6%
	2	T_2_	174	34.7%
	3	T_3_	80	15.9%
	4	T_4_	76	15.1%
	9	T_X_	139	27.7%
Nodal involvement	0	N_0_	140	27.9%
	1	N_1_	166	33.1%
	2	N_2_	43	8.6%
	3	N_3_	4	8.0%
	9	N_X_	149	29.7%

FNAC: Fine needle aspiration cytology; BCT: Breast conservation treatment; MRM: Modified radical mastectomy.

The over all mean (±SD) quality of life score was 90.5 (±18.4) (median 87) ranging from 38–136.5. Mean score for various subscales were: physical well-being (GP) 19.8 ± 4.7; social family well-being (GS) 19.9 ± 5.3; Emotional well-being (GE) 14 ± 14.9 and functional well-being (GF) 13 ± 5.7. The mean scores for breast subscale was 23.07 ± 4.3 (median 24.8 range 10–34.7). The median and score range is detailed in table 3. The mean (±SD) subscale and scale scores for various variables are detailed in additional tables 1 and 2.

**Table 3:** Mean, standard deviation, median and range of the QOL scale, subscale scores and patient age.

Parameter	Mean ± SD	Median	Range
Age	47.65 ± 11	47	20–80
General Physical well-being (GP)	19.8 ± 4.7	20	3–28
General Social well-being (GS)	19.9 ± 5.3	20.5	2.8–28
General Emotional well-being (GE)	14 ± 4.9	14	1–24
General Functional well-being (GF)	13 ± 5.7	12	2–28
Breast specific subscale (B)	23.7 ± 4.3	24.8	10–34.7
Total FACT-B score	90.5 ± 18.4	87	38–136.5

On univariate analysis, patient's education (p = 0.004), spouse occupation (0.01), number of children (p = 0.02), previous treatment (p = 0.02), nodal stage (p = 0.03), metastasis (p = 0.000) and composite stage (p = 0.000) of the disease were found to influence physical well-being (additional file 1).

The distance travelled to reach the treatment centre (p = 0.04), religion of the patient (p = 0.006) marital status (p = 0.002), education (p = 0.04) self (p = 0.02) and spouse occupation (0.04), method of diagnosis (p = 0.000), previous treatment (p = 000) and nodal status (p = 0.02) were found to significant influence emotional well-being.

Functional well-being was found to be influenced by religion (p = 0.000), patients education (p = 0.000), self (p = 0.000) and spouse occupation (p = 0.001), mode of diagnosis (p = 0.01), previous treatment (p = 0.02), and nodal status (p = 0.01). While distance travelled to the centre (p = 0.003) patients education, mode of diagnosis, previous treatment, presence of metastasis and composite stage significantly influenced breast specific subscale.

The overall quality of life was found be significantly affected by income (p = 0.03), Religion (p = 0.005), patients education (p = 0.000), self (p = 0.004) and spouse occupation (p = 0.000) presence of pain (p = 0.001), method of diagnosis (p = 0.000), previous treatment (p = 0.02), nodal stage (p = 0.01), presence of metastasis (p = 0.04) and composite stage (p = 0.005) (additional file 1).

### Result of multiple logistic regression

**Figure 1:**
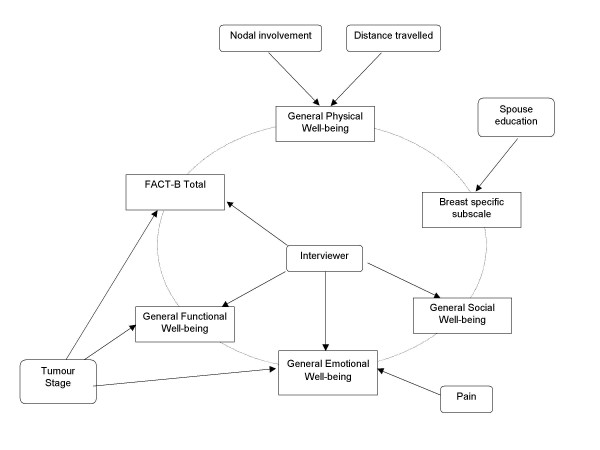
Factors influencing quality of life of women with breast cancer in India identified using logistic regression.

Additional file 2 shows results of multivariate analysis. Distance travelled to the treatment centre and presence of nodal metastasis at initial presentation was found to significantly influence physical well-being. Social and family well-being was affected by religion. Emotional well-being was significantly influenced by religion and tumour stage at presentation. Functional well-being was influenced by religion, presence or absence of pain and tumour stage at presentation. While education of spouse was found to influence breast specific subscale, the overall quality of life was found to be significantly influenced by religion and tumour stage of the disease at presentation (Figure 1).

## Discussion

In India, comprehensive cancer care is provided in the tertiary care centres and due to fewer numbers of such centres there are ever increasing patient load on each of them. Most patients present in locally advanced stage and achieving a good survival is still a priority. However, a few attempts have been made to comprehend and address the psychological and social needs of cancer patients [8,18,19].

The state of Kerala has a unique distinction of being 90% literate and having more females than males in the society [20]. However, as the state offers few employment opportunities, the per-capita income is low, and migration to other states and countries is high. It is also seen as a borrower's economy and is often termed as a consumer state. All these factors contribute to the state's high cost of living despite poor average earnings. Hence, developing a chronic illness or having a spouse with chronic illness like cancer would mean loss of that day's income, and extra expenditures. This reflects in the present study as well as the family income was found to significantly influence the overall quality of life.

Initial diagnosis has been shown to evoke a state of shock, fear and disbelief [21] thus creating not only a psychological crisis but an existential one as well [22]. Education has been found to significantly help one cope with these situations. In the present study too, the education was found to be a significant predictor of overall QOL in univariate analysis, however this significance was lost in the multivariate analysis. Spouse education was found to significantly influence social well-being in the univariate analysis, however in multivariate analysis it was found to significantly influence the breast specific subscale.

Culturally, Indian parents are substantially involved in their offspring's personal and social development, education, and more importantly their marriage, as majority of the marriages are arranged. Such marriages are stressful particularly for the parents of girls. Issues around dowry, sometimes described as a "social evil", play a significant determining role in marriage alliances. Adding the taboo of a parent with cancer affords even greater psychological pressure and financial burden on a family with unmarried children. This is more in patients with lower and middle income where the resources are meagre. The diagnosis of a cancer in the family also has its social stigma, which may influence the marriage prospects of the children. This is reflected in the results of the present study where number of unmarried children was found to significantly affect emotional well-being.

The present study has identified several factors that influence the QOL of the Indian breast cancer patient. Presence of pain has been identified to significantly influence physical well-being and overall QOL, stage of disease has been identified to influence functional well-being and breast specific subscale. In the univariate analysis, the distance travelled by a patient to the treatment centre significantly influenced the breast specific QOL and emotional well-being, however in the multivariate analysis it was found to significantly influence only the patient's physical well-being as expected. It was also interesting to note that though the univariate analysis did not indicate 'tumour stage' as an indicator of QOL in the breast cancer patient, the multivariate analysis showed its significant influence on emotional and functional well-being as well as on over all QOL score. In contrast to this, 'nodal involvement' was noted to influence the physical, emotional, functional well-being, and overall QOL score in the univariate analysis, but was found to significantly influence only the patients physical well-being in multivariate analysis. Several other variables that were found to have significant effect on quality of life and subscales in the univariate analysis turned out as insignificant in the multivariate analysis, viz. gender of the interviewer, and patient occupation etc.

The need for psychosocial intervention amongst cancer patients cannot be understated. The goals of planning a psychosocial intervention in the Indian breast cancer context would be to support the patient's ability to cope with the stress of treatment, helping them to tolerate short-term loss for long-term gain, and to assist in symptom management [21,24-26]. However, owing to increased patient burden, in-depth psychological intervention to each patient may not be feasible, and some sort of mechanism to cater to psychosocial problems need to be identified. Identification of the subset of women at risk is one such way forward, followed by targeted intervention that could be in form of patient education and counselling.

## Competing interests

MP is the editor-in-chief of *World Journal of Surgical Oncology*, published by Open Access publishers Biomedcentral, which depends on Open Access model for substantial portion of its revenue.

## Ethical approval

The study is approved by the institutional review board and the ethics committee.

## Authors' contributions

**MP**: designed and coordinated the study, participated in statistical analysis, helped in preparing the draft manuscript and edited the final version for publication, beside contribution to patient management.

**BCT**: Participated in the study, data collection and statistical analysis and drafted the manuscript

**PS**: Participated in data collection and preparation of the manuscript

**KR, KR, SP, BSM and BR **contributed in patient management, study design and conduct and interpretation of results. They also contributed to the intellectual content of the manuscript.

All the authors read and approved the final version of the manuscript for publication

**MP and KR **are the guarantors of the manuscript

## Supplementary Material

Additional file 1 QOL Breast 2005 showing results of univariate analysis. Click here for file

Additional file 2 QOL breast 2005 showing results of multivariate analysis, multivariate odds ratios and p values. Click here for file
