# Supplementary material for: Quality of Life determinants in women with breast cancer undergoing treatment with curative intent
Source: World J Surg Oncol. 2005 Sep 27;3:63. doi: 10.1186/1477-7819-3-63 (PMC1261539; doi:10.1186/1477-7819-3-63)
Supplement: Additional file 1 — QOL Breast 2005 showing results of univariate analysis. [file 1477-7819-3-63-S1.doc]

# Additional file 1: Results of univariate analysis

| **Variable** | **GP**  **F (p)** | **GS**  **F (p)** | **GE**  **F (p)** | **GF**  **F (p)** | **B**  **F (p)** | **Total**  **F (p)** |
| --- | --- | --- | --- | --- | --- | --- |
| ***Interviewer*** | 27.2 (0.000)* | 238 (0.000)* | 107 (0.000)* | 306 (0.000)* | 0.1 (0.6) | 190 (0.000)* |
| ***Income*** | 2.3 (0.06) | 2.4 (0.06) | 2.2 (0.08) | 1.3 (0.24) | 2.1 (0.8) | 2.9 (0.03)*  low VS High# |
| ***Distance travelled*** | 1.5 (0.2) | 0.2 (0.9) | 2.4 (0.04)* | 0.5 (0.7) | 4.0 (0.003)* | 1.7 (0.14) |
| ***Religion*** | 0.6 (0.5) | 1.4 (0.2) | 4.2 (0.006)*  1&2 with 3 | 8.0 (0.000)*  1&2 with 3 | 0.8 (0.4) | 4.3 (0.005)*  1&2 with 3 |
| ***Marital status*** | 1.9 (0.1) | 1.8 (0.1) | 5.1 (0.002)* | 1.2 (0.29) | 0.06 (0.9) | 2.0 (0.11) |
| ***Patient education*** | 2.8 (0.004)* | 2.6 (0.009)* | 2 (0.04)* | 3.7 (0.000)* | 2 (0.04)* | 3.9 (0.000)* |
| ***Spouse Education*** | 0.9 (0.5) | 2.2 (0.04)* | 1.6 (0.13) | 1.6 (0.12) | 0.2 (0.9) | 1.5 (0.16) |
| ***Patient occupation*** | 1.3 (0.2) | 3.2 (0.02)*  1 with 2 | 4.9 (0.02)*  2 with 3&9 | 8.2 (0.000)*  1 with 2&9  2 with 3 | 0.6 (0.5) | 4.4 (0.004)*  1 with 2 |
| ***Spouse occupation*** | 3.7 (0.01)*  2 with 9 | 4.9 (0.002)*  2 with 3&9 | 4.5 (0.04)*  2 with 3 | 6.0 (0.001)*  2 with 3&9 | 0.6 (0.5) | 5.9 (0.001)*  2 with 3&9 |
| ***Symptoms*** | 1.9 (0.11) | 5.7 (0.001)* | 2.8 (0.03)* | 7.0 (0.000)* | 3.8 (0.009)* | 6 (0.000)* |
| ***Pain*** | 12.5 (0.000)* | 2.9 (0.05)* | 2.6 (0.07) | 4.5 (0.01)* | 0.8 (0.4) | 6.7 (0.001)* |
| ***Mode of diagnosis*** | 2 (0.1) | 5.5 (0.001)*  1 with 2 | 6.9 (0.000)*  1 with 2 | 10.8 (0.000)*  1 with 2 | 3.5 (0.01)*  1 with 2 | 7.6 (0.000)*  1 with 2 |
| ***Previous treatment*** | 2.8 (0.02)* | 1.0 (0.37) | 6.2 (0.000)* | 1.6 (0.14)* | 3 (0.02)* | 2.8 (0.02)* |
| ***Children*** | 3.9 (0.02)* | 0.7 (0.4) | 0.15 (0.8) | 1.2 (0.3) | 0.4 (0.6) | 0.8 (0.4) |
| ***Married Children*** | 2.1 (0.1) | 0.6 (0.5) | 1.2 (0.2) | 2.1 (0.1) | 1.7 (0.1) | 0.8 (0.4) |
| ***Tumour stage (T)*** | 0.9 (0.4) | 0.1 (0.9) | 0.2 (0.8) | 0.1 (0.9) | 1.1 (0.3) | 0.2 (0.8) |
| ***Node stage (N)*** | 3.0 (0.03)*  0 with 1 | 1.7 (0.14) | 3.1 (0.02)*  0 with 1 | 3.6 (0.01)*  0 with 1 | 0.6 (0.5) | 3.8 (0.01)*  0 with 1 |
| ***Metastasis (M)*** | 18.1 (0.000)* | 0.1 (0.7) | 2.1 (0.14) | 0.6 (0.4) | 3.7 (0.05)* | 4.1 (0.04)* |
| ***Stage Composite*** | 7.7 (0.000)* | 0.9 (0.4) | 2.3 (0.07) | 2 (0.1) | 2.7 (0.05)* | 4.2 (0.005)* |

GP- Physical well-being; GS- Social and family well-being; GE- Emotional well-being; GF- Functional well-being; B- Breast specific subscale; Total- Overall FACT-B score. F- Oneway ANOVA value; p- Probability; * Significant.
