# Supplementary material for: Quality of Life determinants in women with breast cancer undergoing treatment with curative intent
Source: World J Surg Oncol. 2005 Sep 27;3:63. doi: 10.1186/1477-7819-3-63 (PMC1261539; doi:10.1186/1477-7819-3-63)
Supplement: Additional file 2 — QOL breast 2005 showing results of multivariate analysis, multivariate odds ratios and p values. [file 1477-7819-3-63-S2.doc]

Additional file 2: Multivariate analysis showing multivariate odds ratio and the p values

| Variable |  | **GP** |  | **GS** |  | **GE** |  | **GF** |  | B |  | **Fact** | **Total** |
| --- | --- | --- | --- | --- | --- | --- | --- | --- | --- | --- | --- | --- | --- |
|  |  | OR | P | OR | P | OR | P | OR | P | OR | P | OR | P |
| ***Children*** | 1 | 0.61 | 0.27 | 0.64 | 0.31 | 0.56 | 0.19 | 0.64 | 0.32 | 1.5 | 0.26 | 0.43 | 0.07 |
|  | 2 | 1.1 | 0.73 | 1.02 | 0.93 | 0.79 | 0.49 | 1.0 | 0.98 | 0.8 | 0.49 | 1.2 | 0.58 |
|  | 3 | 1.0 | - | 1.0 | - | 1.0 | - | 1.0 | - | 1.0 | - | 1.0 | - |
| ***Metastasis*** | 0 | α | 0.58 | 1.87 | 0.27 | 2.1 | 0.2 | 0.85 | 0.81 | α | 0.72 | 2.15 | 0.28 |
|  | 1 | 1.0 | - | 1.0 | - | 1.0 | - | 1.0 | - | 1.0 | - | 1.0 | - |
| ***Married child*** | 0 | 1.37 | 0.45 | 1.44 | 0.38 | 1.15 | 0.7 | 1.0 | 0.97 | 0.85 | 0.70 | 1.0 | 0.97 |
|  | 1 | 1.16 | 0.65 | 1.5 | 0.22 | 1.77 | 0.12 | 1.39 | 0.37 | 1.03 | 0.92 | 1.8 | 0.11 |
|  | 2 | 1.0 | - | 1.0 | - | 1.0 | - | 1.0 | - | 1.0 | - | 1.0 | - |
| ***Node*** | 0 | 3.2 | 0.03* | 1.0 | 0.94 | 19.7 | 0.72 | 28.5 | 0.81 | 1.5 | 0.44 | 1.43 | 0.57 |
|  | 1 | 0.92 | 0.87 | 1.3 | 0.6 | 13.1 | 0.76 | 12.7 | 0.85 | 0.78 | 0.61 | 0.96 | 0.94 |
|  | 2 | 0.49 | 0.37 | 0.58 | 0.45 | 4.2 | 0.86 | 5.4 | 0.9 | 1.02 | 0.96 | 1.31 | 0.73 |
|  | 3 | 1.0 | - | 1.0 | - | 1.0 | - | 1.0 | - | 1.0 | - | 1.0 | - |
| ***Pain*** | 1 | 1.32 | 0.61 | 1.29 | 0.59 | 1.6 | 0.40 | 4.6 | 0.02* | 0.85 | 0.80 | 1.2 | 0.72 |
|  | 2 | 1.54 | 0.42 | 1.59 | 0.31 | 2.27 | 0.15 | 4.17 | 0.03* | 0.88 | 0.75 | 2.2 | 0.19 |
|  | 9 | 1.0 | - | 1.0 | - | 1.0 | - | 1.0 | - | 1.0 | - | 1.0 | - |
| ***Religion*** | 1 | 1.0 | 0.96 | 0.73 | 0.43 | 1.66 | 0.23 | 1.4 | 0.49 | 0.81 | 0.59 | 1.2 | 0.59 |
|  | 2 | 0.83 | 0.72 | 0.81 | 0.68 | 0.54 | 0.27 | 0.8 | 0.73 | 0.43 | 0.11 | 0.34 | 0.06 |
|  | 3 | 0.83 | 0.72 | 3.13 | 0.04* | 5.49 | 0.003* | 4.8 | 0.02* | 1.38 | 0.5 | 6.9 | 0.003* |
|  | DK | 1.0 | - | 1.0 | - | 1.0 | - | 1.0 | - | 1.0 | - | 1.0 | - |
| ***T*** | 1 | 0.89 | 0.8 | 0.5 | 0.16 | 0.3 | 0.01* | 0.28 | 0.02* | 0.57 | 0.26 | 0.28 | 0.02* |
|  | 2 | 0.7 | 0.43 | 1.36 | 0.37 | 1.48 | 0.28 | 0.84 | 0.64 | 1.13 | 0.71 | 1.12 | 0.74 |
|  | 3 | 1.1 | 0.8 | 0.94 | 0.87 | 0.95 | 0.90 | 0.69 | 0.43 | 1.18 | 0.65 | 1.85 | 0.17 |
|  | 4 | 1.0 | - | 1.0 | - | 1.0 | - | 1.0 | - | 1.0 | - | 1.0 | - |
| ***Category*** | 1 | 1.2 | 0.56 | 0.91 | 0.77 | 0.76 | 0.43 | 0.84 | 0.64 | 1.02 | 0.94 | 0.77 | 0.46 |
|  | 2 | 0.78 | 0.44 | 0.75 | 0.36 | 0.87 | 0.68 | 0.58 | 0.1 | 1.02 | 0.93 | 0.67 | 0.22 |
|  | 3 | 1.0 | - | 1.0 | - | 1.0 | - | 1.0 | - | 1.0 | - | 1.0 | - |
| ***District*** | 1 | 1.4 | 0.28 | 1.4 | 0.34 | 1.32 | 0.46 | 1.5 | 0.3 | 1.7 | 0.15 | 1.25 | 0.56 |
|  | 2 | 1.8 | 0.11 | 1.3 | 0.45 | 1.3 | 0.47 | 0.8 | 0.57 | 1.2 | 0.56 | 0.81 | 0.59 |
|  | 3 | 0.29 | 0.02* | 1.1 | 0.85 | 0.82 | 0.71 | 2.0 | 0.17 | 0.44 | 0.14 | 1.6 | 0.36 |
|  | 4 | 1.0 | - | 1.0 | - | 1.0 | - | 1.0 | - | 1.0 | - | 1.0 | - |
| ***Pt. Edu*** | 0 | 5.3 | 0.93 | 0.13 | 0.91 | 0.38 | 0.93 | 0.01 | 0.81 | 2.54 | 0.96 | 0.45 | 0.94 |
|  | 1 | 5.2 | 0.93 | 1.4 | 0.98 | 0.39 | 0.93 | 0.06 | 0.88 | 0.74 | 0.98 | 0.24 | 0.90 |
|  | 2 | 17.2 | 0.88 | 0.85 | 0.99 | 0.47 | 0.94 | 0.12 | 0.91 | 3.7 | 0.94 | 0.62 | 0.96 |
|  | 3 | 3.6 | 0.94 | 0.21 | 0.93 | 0.21 | 0.89 | 0.03 | 0.85 | 1.5 | 0.98 | 0.22 | 0.89 |
|  | 4 | 6.4 | 0.92 | 0.44 | 0.96 | 0.98 | 0.99 | 0.18 | 0.93 | 2.6 | 0.96 | 2.67 | 0.93 |
|  | 5 | 2.2 | 0.96 | 1.3 | 0.98 | 0.55 | 0.95 | 0.09 | 0.90 | 0.96 | 0.99 | 1.2 | 0.98 |
|  | DK | 1.0 | - | 1.0 | - | 1.0 | - | 1.0 | - | 1.0 | - | 1.0 | - |
| ***Sp. Edu*** | 0 | 0.1 | 0.77 | 0.2 | 0.25 | 0.31 | 0.34 | 0.00 | 0.79 | 0.32 | 0.36 | 0.16 | 0.22 |
|  | 1 | 1.13 | 0.98 | 1.4 | 0.56 | 1.0 | 0.97 | 3.4 | 0.80 | 3.9 | 0.03* | 1.4 | 0.57 |
|  | 2 | 0.16 | 0.81 | 0.38 | 0.07 | 0.88 | 0.82 | 1.6 | 0.91 | 1.11 | 0.82 | 1.0 |  |
|  | 3 | 0.09 | 0.75 | 1.9 | 0.55 | 3.7 | 0.24 | 16.6 | 0.56 | 0.45 | 0.42 | 0.96 | 0.9 |
|  | 4 | 0.70 | 0.96 | 4.0 | 0.09 | 3.21 | 0.15 | 8.8 | 0.65 | 1.18 | 0.83 | 3.9 | 0.97 |
|  | 5 | 0.33 | 0.88 | 2.9 | 0.36 | 1.0 | 0.99 | 2.2 | 0.86 | 3.73 | 0.21 | 3.9 | 0.12 |
|  | DK | 1.0 | - | 1.0 | - | 1.0 | - | 1.0 | - | 1.0 | - | 1.0 | - |

GP- Physical well-being; GS- Social and family well-being; GEMO- Emotional well-being; GF- Functional well-being; B- Breast specific subscale; Total- Overall FACT-B score; DK- Don’t know; * Significant, OR- odds ratio, P- probability value.
